# Postoperative infectious complications following laparoscopic versus open hepatectomy for hepatocellular carcinoma: a multicenter propensity score analysis of 3876 patients

**DOI:** 10.1097/JS9.0000000000000446

**Published:** 2023-05-10

**Authors:** Jia-Le Pu, Xiao Xu, Lan-Lan Chen, Chao Li, Hang-Dong Jia, Zhong-Qi Fan, Ju-Dong Li, Ming-Cheng Guan, Ying-Jian Liang, Ya-Hao Zhou, Xian-Ming Wang, Wei-Min Gu, Hong Wang, Jie Li, Zhi-Yu Chen, Ting-Hao Chen, Yao-Ming Zhang, Zi-Xiang Chen, Lan-Qing Yao, Yong-Kang Diao, Ming-Da Wang, Feng Shen, Timothy M. Pawlik, Wan Yee Lau, Zhong Chen, Tian Yang, Guo-Yue Lv

**Affiliations:** a Department of Hepatobiliary and Pancreatic Surgery, General Surgery Center, First Hospital of Jilin University, Changchun, Jilin; b Department of Hepatobiliary Surgery, Affiliated Hospital of Nantong University, Nantong; c Department of Medical Oncology, the First Affiliated Hospital of Soochow University, Soochow, Jiangsu; d Department of General Surgery, Cancer Center, Division of Hepatobiliary and Pancreatic Surgery, Zhejiang Provincial People’s Hospital, Affiliated People’s Hospital, Hangzhou Medical College, Hangzhou, Zhejiang; e Department of Pancreatic-biliary Surgery, Changzheng Hospital; f Department of Hepatobiliary Surgery, Eastern Hepatobiliary Surgery Hospital, Second Military Medical University (Navy Medical University), Shanghai; g Department of Hepatobiliary Surgery, the First Affiliated Hospital of Harbin Medical University; h The First Department of General Surgery, the Fourth Hospital of Harbin, Harbin, Heilongjiang; i Department of General Surgery, First Affiliated Hospital of Shandong First Medical University & Shandong Provincial Qianfoshan Hospital, Jinan, Shandong; j Department of Hepatobiliary Surgery, Pu’er People’s Hospital, Pu’er, Yunnan; k Department of General Surgery, Liuyang People’s Hospital, Liuyang, Hunan; l Department of Hepatobiliary Surgery, Fuyang People’s Hospital, Fuyang; m Department of General Surgery, The First Affiliated Hospital of Anhui Medical University, Hefei, Anhui; n Department of General Surgery, Ziyang First People’s Hospital, Ziyang, Sichuan; o The Second Department of Hepatobiliary Surgery, Meizhou People’s Hospital, Meizhou, Guangdong; p Department of Hepatobiliary Surgery, Southwest Hospital, Third Military Medical University (Army Medical University), Chongqing; q Faculty of Medicine, the Chinese University of Hong Kong, Shatin, New Territories, Hong Kong SAR, China; r Department of Surgery, The Ohio State University Wexner Medical Center and James Comprehensive Cancer Center, Columbus, Ohio, USA

**Keywords:** hepatectomy, hepatocellular carcinoma, morbidity, operative approach, postoperative complications, surgical site infection

## Abstract

**Objectives:**

Hepatocellular carcinoma (HCC) is a common indication for hepatectomy that is often complicated by postoperative complication. The authors sought to investigate the relationship between the open with laparoscopic approach of hepatectomy and incidences of postoperative infectious complications.

**Patients and methods:**

Using a multicenter database, HCC patients who underwent laparoscopic hepatectomy (LH) or open hepatectomy (OH) were reviewed and analyzed. Propensity score matching (PSM), inverse probability of treatment weight (IPTW), and multivariate logistic regression analyses were utilized to assess the association of the operative approach with postoperative infectious complications, including incisional surgical site infection (SSI), organ/space SSI, and remote infection (RI).

**Results:**

Among 3876 patients, 845 (21.8%) and 3031 (78.2%) patients underwent LH and OH, respectively. The overall incidence of infection was 6.9 *versus* 14.6% among patients who underwent LH *versus* OH, respectively (*P*<0.001). Of note, the incidences of incisional SSI (1.8 vs. 6.3%, *P*<0.001), organ/space SSI (1.8 vs. 4.6%, *P*<0.001), and RI (3.8 vs. 9.8%, *P*<0.001) were all significantly lower among patients who underwent LH *versus* OH. After PSM (6.9, 1.8, 1.8, and 3.8% vs. 18.5, 8.4, 5.2, and 12.8%, respectively) and IPTW (9.5, 2.3, 2.1, and 5.5% vs. 14.3, 6.3, 4.5, and 9.8%, respectively), LH remained associated with statistically lower incidences of all types of infectious complications. After adjustment for other confounding factors on multivariate analyses, LH remained independently associated with lower incidences of overall infection, incisional SSI, organ/space SSI, and RI in the overall, PSM, and IPTW cohorts, respectively.

**Conclusion:**

Compared with open approach, laparoscopic approach was independently associated with lower incidences of postoperative infectious complications following hepatectomy for HCC.

## Graphical abstract

**Figure:**
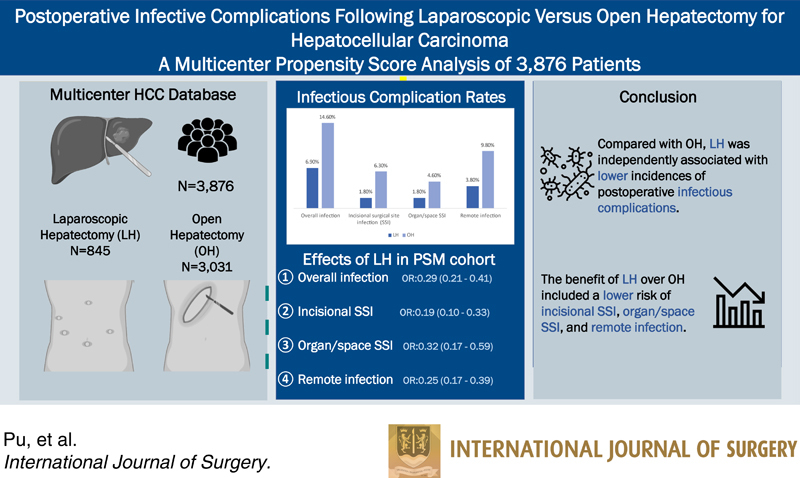

## Introduction

Highlights

- Compared with open hepatectomy, laparoscopic hepatectomy was independently associated with lower incidences of postoperative infectious complications following hepatectomy for hepatocellular carcinoma.
- The benefit of laparoscopic hepatectomy over open hepatectomy included a lower risk of incisional surgical site infection, organ/space surgical site infection, and remote infection.

Hepatectomy remains the curative-intent treatment modality of choice for patients with hepatocellular carcinoma (HCC)^1,2^. With improvement in surgical techniques, operative devices, and perioperative care, postoperative mortality following hepatectomy has been reduced to less than 5%, and even below 1% in some experienced centers^3,4^. Unfortunately, the incidence of postoperative complications remains high ranging from 20 to 70%^5–8^. In turn, postoperative complications are independently associated with prolonged hospital stay, increased hospital costs, and a higher risk of early death^8–11^. Among all posthepatectomy complications, postoperative infection, including incisional surgical site infection (SSI), organ/space SSI, and remote infection (RI), are among the most common^12–17^. Posthepatectomy infectious complications can require surgical debridement(s), increase the risk of postoperative hepatic failure, as well as worsen long-term oncological survival after HCC resection^18–20^. As such, identifying and reducing risk factors associated with postoperative infectious complications are critical to improve short-term and long-term outcomes among patients undergoing hepatectomy for HCC.

Although open hepatectomy (OH) is still the primary operative approach for hepatectomy in most hospitals worldwide, laparoscopic hepatectomy (LH) has been increasingly used at many high-volume hepatobiliary centers. LH offers the potential advantages of fewer overall postoperative complications, a shorter hospital stay, and better postoperative cosmetic satisfaction^21–24^. However, few studies have specifically examined the impact of the operative approach relative to the incidence of posthepatectomy infectious complications^25–28^. Moreover, these few studies were all carried out at a single center and had a limited sample size^25–28^, with some reports only including univariate but not multivariate analysis^25,27,28^; other reports only evaluated one specific type of postoperative infectious complication, such as SSI^27,28^ or pulmonary infection^26^, but not the overall incidence of all types of infectious complications.

Therefore, the objective of the current study was to evaluate the impact of the operative approach on the incidences of different types of postoperative infectious complications following hepatectomy for HCC. To minimize selection bias and the effect of confounding factors, two propensity scoring methods and multivariate logistic regression analyses were utilized to analyze the data from a large multicenter database.

## Patients and methods

### Study population

A multicenter database on consecutive patients who underwent curative-intent hepatectomy for HCC from 2010 to 2021 at 16 hospitals in China was retrospectively reviewed and analyzed. The inclusion criteria were patients who: had HCC confirmed by histopathological examination in the resected specimen; had no previous anti-HCC treatment (i.e. transcatheter arterial chemoembolization, portal vein embolization, and associating liver partition and portal vein ligation for staged hepatectomy) before hepatectomy. The exclusion criteria were patients who: had combined HCC and cholangiocarcinoma; had recurrent or ruptured HCC; underwent concomitant biliary reconstruction or gastrointestinal surgical procedures during hepatectomy; had an acute attack of bacterial infection within 2 weeks prior to hepatectomy; and underwent conversion from laparoscopic to OH. Informed consent for the data to be used for clinical research was obtained from all patients. The study was conducted in accordance with the Declaration of Helsinki and the Ethical Guidelines for Clinical Studies and was approved by the Institutional Review Boards at the participating hospitals. This retrospective study was registered with ResearchRegistry.com. Data has been reported in line with the strengthening the reporting of cohort, cross-sectional and case-control studies in surgery (STROCSS) 2021 criteria^29^, Supplemental Digital Content 1, http://links.lww.com/JS9/A464.

### Clinical characteristics and operative variables

Patient clinical characteristics included age, sex, BMI, diabetes mellitus, American Society of Anesthesiologists (ASA) score, hepatitis B virus (HBV) infection status, hepatitis C virus (HCV) infection status, cirrhosis, portal hypertension, Child-Pugh grading, maximum tumor size, tumor number, and gross vascular invasion. Operative variables included operative approach (OH or LH), operation period (the first period: 2010–2015 or the second period: 2016–2021), extent of hepatectomy (major or minor), intraoperative blood loss, and intraoperative blood transfusion. Obesity was defined as a BMI greater than or equal to 30 kg/m^2^. Portal hypertension was defined as the presence of splenomegaly with a decreased platelet count (≤ 100×10^9^/l) and/or esophageal varices. Major hepatectomy was defined as partial hepatectomy of three or more Couinaud’s liver segments, while minor hepatectomy was defined as fewer than three segments.

### Surgical procedures and perioperative management

Perioperative evaluation and surgical procedures were generally consistent at each hospital; specifically, the criteria for hepatectomy were constant over the study period. The technical details of OH have been described previously^30,31^. LH was performed under CO_2_ pneumoperitoneum with pressure maintained at 12–15 mmHg. Four or five trocars were usually inserted. Laparoscopic ultrasound or fluorescence navigation was used to detect tumor and vascular -related conditions. Perihepatic ligaments and hepatic parenchyma were transected by a harmonic scalpel or a Cavitron Ultrasonic Surgical Aspirator. Intrahepatic vessels and bile ducts larger than 5 mm were clipped with plastic or titanium clips. Bipolar electrocoagulation and sutures were used for hemostasis. All laparoscopic procedures were performed by surgeons who had professional trainings at Chinese provincial-level teaching hospitals and sufficient experience with other laparoscopic surgery (more than 100 laparoscopic cholecystectomies). Specimens were removed through an extended trocar incision. One abdominal drain was placed in both OH and LH operations in select cases.

Broad-spectrum antibiotics were administered intravenously for 1–3 days. Fresh frozen plasma or albumin was given if the plasma albumin level was lower than 30 g/l. Blood tests and serologic liver function tests were performed 1-, 3-, 5-, and 7 days after resection. All patients underwent chest radiograph examinations on postoperative day (POD) 3 and ultrasound during the first week after resection. Blood transfusions were administered to patients whose hemoglobin level was below 7 mg/dl. As a general rule, drains were removed when no bleeding, bile leak, or massive drainage of ascites was observed on POD 2–4.

### Postoperative outcomes

Postoperative mortality was defined as death within 30 days of surgery and the causes of mortality were recorded. Postoperative morbidity was standardized as occurring within 30 days of surgery and graded according to the Clavien–Dindo classification^32^. Minor morbidity was defined as Clavien–Dindo I–II and major morbidity was defined as Clavien–Dindo III–V. Postoperative complications were further categorized as infectious or noninfectious; the former included SSI and RI, whereas the latter included hepatic dysfunction, abdominal hemorrhage, bile leak, ascites, pleural effusion, and other complications. SSI within 30 days of surgery was diagnosed according to the Centers for Disease Control and Prevention of the National Nosocomial Infections Surveillance, which were classified as either incisional SSI or organ/space SSI^33^. RI was defined as an infection at locations or organs distant from any surgical fields, including respiratory infection, gastrointestinal tract infection, urinary tract infections, catheter-related bloodstream infections, and systemic sepsis^25^. Postoperative hepatic dysfunction was defined according to the ‘50–50 criteria’ on or after POD 5^34^. Abdominal hemorrhage was defined by a drop in hemoglobin level of more than 3 g/dl compared with the preoperative baseline level and/or any postoperative transfusion of packed red blood cells units for decreasing hemoglobin level and/or the need for invasive reintervention^35^. A bile leak was diagnosed based on a drain bilirubin concentration more than threefold higher than that in serum^36^. Ascites and pleural effusion that required diuretics or paracentesis were also included as morbidity^37^.

### Statistical analysis

Patients were divided into two cohorts based on operative approach: OH and LH. Categorical variables were expressed as number (*n*) or proportion (%), while continuous variables were expressed as mean±SD or median (range). Categorical variables were compared using the χ^2^-test or the Fisher’s exact test, as appropriate. The student’s *t*-test was used to compare continuous variables when applicable, otherwise, the Mann–Whitney *U*-test was applied. Postoperative outcomes, particularly the incidence of overall infection and various infectious complications, were compared among patients who underwent OH versus LH.

In order to balance differences in baseline characteristics due to possible selection bias among patients who underwent OH versus LH, two propensity score methods were utilized: propensity score matching (PSM) and inverse probability of treatment weight (IPTW). Variables entered into the propensity model included age, sex, BMI, diabetes mellitus, ASA score, HBV infection status, HCV infection status, cirrhosis, portal hypertension, Child-Pugh grading, maximum tumor size, tumor number, gross vascular invasion, operation period, extent of hepatectomy, intraoperative blood loss, and intraoperative blood transfusion. The PSM method was performed as described by Rubin and Rosenbaum^38,39^ and provided a one-to-one matching between the two groups by using a greedy, nearest neighbor matching algorithm. The matching process fully complies with current guidelines^40^, which has also been described in previous studies^30,40,41^. For the IPTW method, a pseudo population was created by weighting the inverse probability of a patient undergoing OH versus LH based on the propensity score^42^. The model preserved the size of the study population and no study participants were dropped, which was advantageous compared with the PSM method.

A level of *P* value<0.05 was considered statistically significant. Apart from the *P* value, standardized mean difference (SMD) was used to measure differences in baseline characteristics among patients who underwent LH versus OH, with SMD less than 0.1 to indicate negligible differences, and between 0.1 and 0.3 to indicate small differences. In order to adjust for other confounding preoperative and intraoperative risk factors, univariate and multivariate logistic regression analyses were performed to identify the real relationship between the operative approach and the incidence of postoperative infectious complications (overall infection, incisional SSI, organ/space SSI, and RI) in the overall, PSM, and IPTW cohorts, respectively. Variables (*P*<0.1) significant in the univariate analyses were entered into a multivariate logistic regression model with backward stepwise selection. Odds ratios and 95% CIs were reported. Statistical analyses were carried out using the IBM SPSS Statistics version 25.0 (SPSS) and R (version 4.2.2) software.

## Results

**Figure 1:**
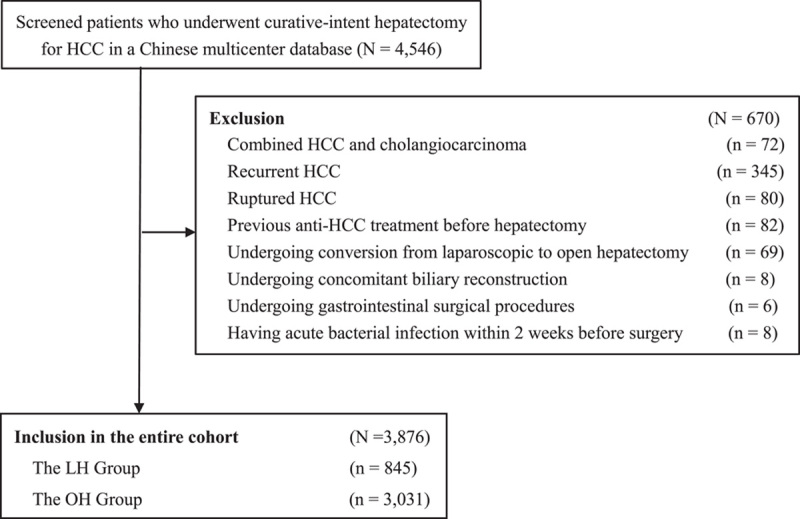
Selection of the study population. HCC, hepatocellular carcinoma; LH, laparoscopic hepatectomy; OH, open hepatectomy.

Among the 4546 patients who underwent curative-intent hepatectomy for HCC during the study period, 3876 patients met inclusion/exclusion criteria and were included in the analytic cohort (Fig. 1). Overall, 845 patients underwent LH (21.8%) and 3031 patients underwent OH (78.2%), respectively. Sixty-nine patients who underwent conversion from laparoscopic to open approach were excluded from the LH cohort. PSM created 845 patient pairs who underwent LH and OH, while IPTW created 3826.6 standardized patients who underwent LH and 3879.9 who underwent OH.

### Comparisons of baseline characteristics

Patient clinical characteristics and operative variables among individuals who underwent OH versus LH are shown in Table 1. Compared with individuals who underwent OH, patients who underwent LH had a higher proportion of individuals age greater than 60 years (42.2 vs. 22.5%, *P*<0.001), diabetes mellitus (14.1 *vs.* 7.5%, *P*<0.001), ASA score greater than 2 (20.5 *vs.* 14.9%, *P*<0.001), and portal hypertension (32.0 *vs.* 23.1%, *P*<0.001), yet a lower proportion of male (82.5 *vs.* 87.7%, *P*<0.001), HBV positiveness (83.2 *vs.* 88.3%, *P*<0.001), HCV positiveness (1.1 *vs.* 2.8%, *P*=0.005), maximum tumor size greater than 5.0 cm (26.3 v*s.* 52.7%, *P*<0.001), multiple tumors (15.3 *vs.* 20.6%, *P*=0.001), gross vascular invasion (5.0 *vs.* 12.3%, *P*<0.001), major hepatectomy (13.6 *vs.* 24.5%, *P*<0.001), intraoperative blood loss greater than 600 ml (14.0 *vs.* 21.4%, *P*<0.001) and intraoperative blood transfusion (17.8 *vs.* 22.2%, *P*=0.006).

**Table 1:** Patients’ clinical characteristics and operative variables in the entire cohort.

*N* (%)	All (*n*=3876)	LH (*n*=845)	OH (*n*=3031)	*P*	SMD
Operation at the second period (2016–2021)	2249 (58.0)	566 (67.0)	1683 (55.5)	<0.001	0.237
Age > 60 years	1038 (26.8)	357 (42.2)	681 (22.5)	<0.001	0.433
Male sex	3355 (86.6)	697 (82.5)	2658 (87.7)	<0.001	0.147
Obesity (BMI ≥ 30.0 kg/m^2^)	124 (3.2)	20 (2.4)	104 (3.4)	0.149	0.063
Diabetes mellitus	347 (9.0)	119 (14.1)	228 (7.5)	<0.001	0.213
ASA score > 2	624 (16.1)	173 (20.5)	451 (14.9)	<0.001	0.147
HBV (+)	3378 (87.2)	703 (83.2)	2675 (88.3)	<0.001	0.145
HCV (+)	95 (2.5)	9 (1.1)	86 (2.8)	0.005	0.128
Cirrhosis	2847 (73.5)	606 (71.7)	2241 (73.9)	0.212	0.050
Portal hypertension	969 (25.0)	270 (32.0)	699 (23.1)	<0.001	0.200
Child-Pugh grade B	359 (9.3)	64 (7.6)	295 (9.7)	0.065	0.077
Maximum tumor size > 5 cm	1818 (46.9)	222 (26.3)	1596 (52.7)	<0.001	0.561
Multiple tumors	752 (19.4)	129 (15.3)	623 (20.6)	0.001	0.138
Gross vascular invasion	414 (10.7)	42 (5.0)	372 (12.3)	<0.001	0.262
Major hepatectomy	858 (22.1)	115 (13.6)	743 (24.5)	<0.001	0.280
Intraoperative blood loss > 600 ml	767 (19.8)	118 (14.0)	649 (21.4)	<0.001	0.196
Intraoperative blood transfusion	822 (21.2)	150 (17.8)	672 (22.2)	0.006	0.111

ASA, American Society of Anesthesiologists; HBV, hepatitis B virus; HCV, hepatitis C virus; LH, laparoscopic hepatectomy; OH, open hepatectomy; SMD, standardized mean difference.

**Figure 2:**
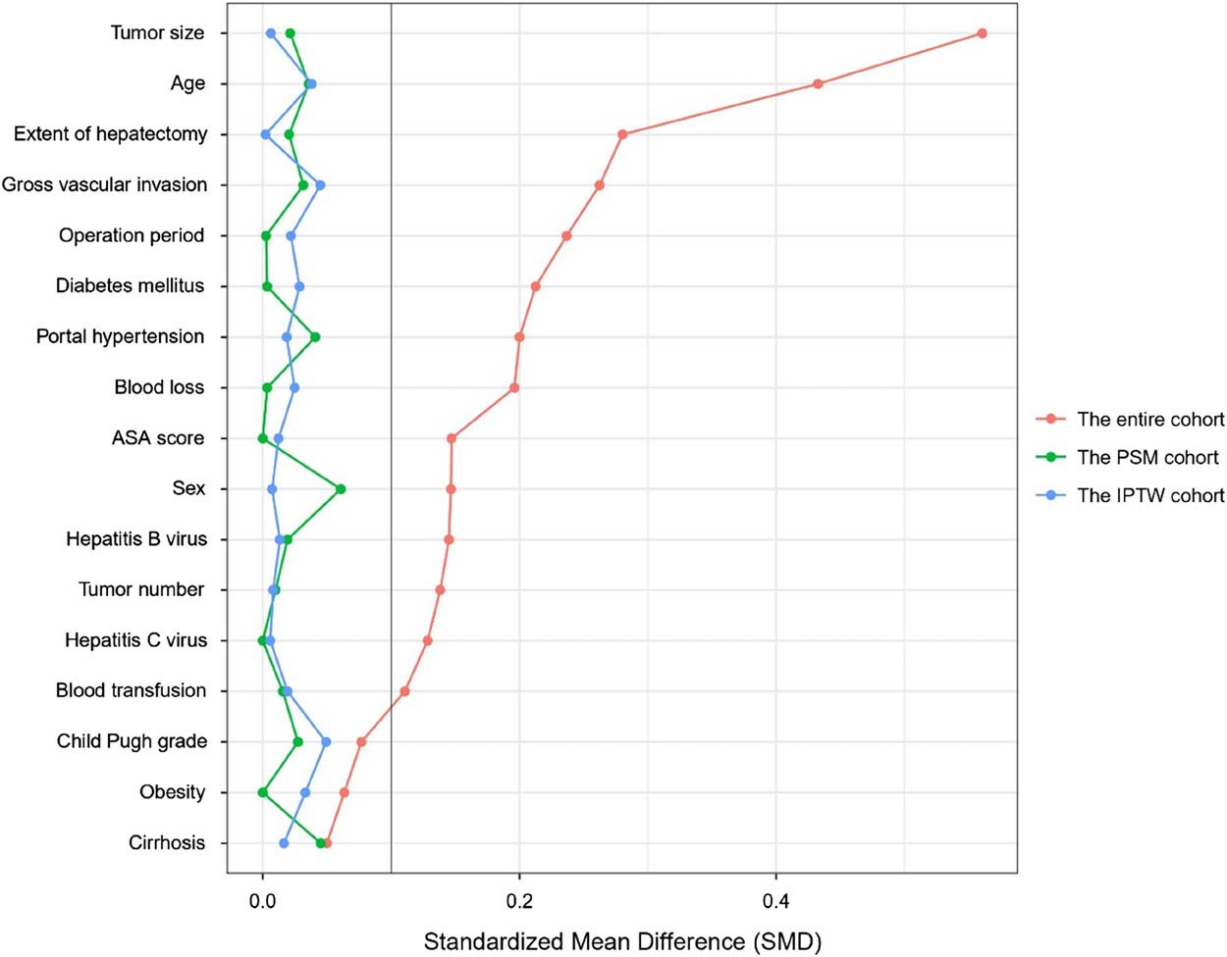
Comparisons of standardized mean difference of clinical variables between laparoscopic and open hepatectomy groups in the entire cohort (red dots), in the PSM cohort (green dots), and in the IPTW cohort (blue dots), respectively. ASA, American Society of Anesthesiologists; IPTW, inverse probability of treatment weight; PSM, propensity score matching.

After applying propensity score analysis, comparisons of clinical characteristics and operative variables of the matched (the PSM cohort) and weighted (the IPTW cohort) study participants are shown in Table 2. Of note, there were no significant differences among patients who underwent OH versus LH for any covariate after matching (all *SMD*<0.1) (Fig. 2).

**Table 2:** Patients’ clinical characteristics and operative variables in the PSM and IPTW cohorts.

	The PSM cohort				The IPTW cohort			
*N* (%)	LH (*n*=845)	OH (*n*=845)	*P*	SMD	LH (*n*=3826.6)	OH (*n*=3879.9)	*P*	SMD
Operation at the second period (2016–2021)	566 (67.0)	565 (66.9)	1.000	0.003	2265.0 (59.2)	2254.7 (58.1)	0.648	0.022
Age > 60 years	357 (42.2)	372 (44.0)	0.492	0.036	1096.9 (28.7)	1046.0 (27.0)	0.366	0.038
Male sex	697 (82.5)	716 (84.7)	0.237	0.061	3328.1 (87.0)	3364.9 (86.7)	0.862	0.007
Obesity (BMI ≥ 30.0 kg/m^2^)	20 (2.4)	20 (2.4)	1.000	<0.001	145.9 (3.8)	124.3 (3.2)	0.555	0.033
Diabetes mellitus	119 (14.1)	120 (14.2)	1.000	0.003	379.9 (9.9)	352.7 (9.1)	0.503	0.029
ASA score > 2	173 (20.5)	173 (20.5)	1.000	<0.001	638.1 (16.7)	629.4 (16.2)	0.782	0.012
HBV (+)	703 (83.2)	709 (83.9)	0.743	0.019	3317.7 (86.7)	3381.3 (87.1)	0.754	0.013
HCV (+)	9 (1.1)	9 (1.1)	1.000	<0.001	90.6 (2.4)	95.3 (2.5)	0.924	0.006
Cirrhosis	606 (71.7)	623 (73.7)	0.382	0.045	2839.6 (74.2)	2851.1 (73.5)	0.711	0.016
Portal hypertension	270 (32.0)	254 (30.1)	0.430	0.041	990.7 (25.9)	973.0 (25.1)	0.675	0.019
Child-Pugh grade B	64 (7.6)	58 (6.9)	0.638	0.027	411.1 (10.7)	359.5 (9.3)	0.377	0.049
Maximum tumor size > 5 cm	222 (26.3)	230 (27.2)	0.700	0.021	1781.5 (46.6)	1818.4 (46.9)	0.895	0.006
Multiple tumors	129 (15.3)	126 (14.9)	0.892	0.010	729.4 (19.1)	751.8 (19.4)	0.875	0.008
Gross vascular invasion	42 (5.0)	48 (5.7)	0.588	0.032	356.7 (9.3)	413.8 (10.7)	0.455	0.045
Major hepatectomy	115 (13.6)	121 (14.3)	0.726	0.020	851.6 (22.3)	860.1 (22.2)	0.968	0.002
Intraoperative blood loss > 600 ml	118 (14.0)	119 (14.1)	1.000	0.003	796.9 (20.8)	769.3 (19.8)	0.639	0.025
Intraoperative blood transfusion	150 (17.8)	145 (17.2)	0.798	0.016	841.6 (22.0)	822.6 (21.2)	0.704	0.019

ASA, American Society of Anesthesiologists; HBV, hepatitis B virus; HCV, hepatitis C virus; IPTW, inverse probability of treatment weight; LH, laparoscopic hepatectomy; OH, open hepatectomy; PSM, propensity score matching; SMD, standardized mean difference.

### Comparisons of postoperative outcomes

Comparison of postoperative outcomes among patients who underwent LH versus OH in the overall cohort, as well as the PSM and IPTW cohorts are shown in Table 3. In each analytic cohort, the postoperative 30-day morbidity among patients who underwent LH was lower than OH (overall cohort: 16.1 *vs.* 28.0%, *P*<0.001; PSM cohort: 16.1 *vs.* 30.5%, *P*<0.001; and IPTW cohort: 20.1 *vs.* 27.4%, *P*=0.001, respectively). Patients who underwent LH also had lower postoperative 30-day minor morbidity (in the entire cohort: 8.4 *vs.* 19.9%, *P*<0.001; in the PSM cohort: 8.4 *vs.* 22.0%, *P*<0.001; and in the IPTW cohort: 10.1 *vs.* 19.4%, *P*<0.001, respectively), as well as postoperative noninfectious complications (in the entire cohort: 12.0 *vs.* 20.8%, *P*<0.001; in the PSM cohort: 12.0 *vs.* 21.3%, *P*<0.001; and in the IPTW cohort: 15.6 *vs.* 20.2%, *P*=0.028, respectively) compared with individuals who underwent OH group. In addition, the mean postoperative hospital stay following LH was shorter than OH in all three analytic cohorts (in the entire cohort: 9.8±6.1 *vs.* 13.0±6.5 d, in the PSM cohort: 9.8±6.1 *vs.* 13.3±7.7 days, and in the IPTW cohort: 10.4±6.2 *vs.* 13.0±6.6 days, respectively; all *P*<0.001).

**Table 3:** Comparisons of postoperative outcomes in the entire, PSM and IPTW cohorts.

	The entire cohort			The PSM cohort			The IPTW cohort		
	LH (*n*=845)	OH (*n*=3031)	*P*	LH (*n*=845)	OH (*n*=845)	*P*	LH (*n*=3826.6)	OH (*n*=3879.9)	*P*
Postoperative 30-day mortality	6 (0.7)	50 (1.6)	0.063	6 (0.7)	15 (1.8)	0.079	62.7 (1.6)	62.4 (1.6)	0.973
Postoperative 30-day morbidity	136 (16.1)	849 (28.0)	<0.001	136 (16.1)	258 (30.5)	<0.001	767.6 (20.1)	1063.1 (27.4)	0.001
Minor morbidity (Clavien–Dindo I–II)	71 (8.4)	602 (19.9)	<0.001	71 (8.4)	186 (22.0)	<0.001	386.0 (10.1)	753.7 (19.4)	<0.001
Major morbidity (Clavien–Dindo III–V)	65 (7.7)	247 (8.1)	0.719	65 (7.7)	72 (8.5)	0.593	381.6 (10.0)	309.4 (8.0)	0.176
Postoperative infectious complications	58 (6.9)	442 (14.6)	<0.001	58 (6.9)	156 (18.5)	<0.001	363.2 (9.5)	556.0 (14.3)	0.015
Incisional SSI	15 (1.8)	192 (6.3)	<0.001	15 (1.8)	71 (8.4)	<0.001	88.5 (2.3)	242.9 (6.3)	0.001
Organ/space SSI	15 (1.8)	140 (4.6)	<0.001	15 (1.8)	44 (5.2)	<0.001	81.9 (2.1)	173.7 (4.5)	0.032
RI	32 (3.8)	297 (9.8)	<0.001	32 (3.8)	108 (12.8)	<0.001	210.0 (5.5)	379.4 (9.8)	0.018
Respiratory infection	12 (1.4)	74 (2.4)	0.099	12 (1.4)	37 (4.4)	0.001	36.3 (0.9)	111.9 (2.9)	0.001
Gastrointestinal tract infection	4 (0.5)	41 (1.4)	0.054	4 (0.5)	19 (2.2)	0.003	24.3 (0.6)	54.3 (1.4)	0.180
Urinary tract infection	10 (1.2)	83 (2.7)	0.013	10 (1.2)	26 (3.1)	0.012	68.0 (1.8)	99.3 (2.6)	0.421
Catheter-related bloodstream infection	4 (0.5)	36 (1.2)	0.104	4 (0.5)	10 (1.2)	0.180	38.8 (1.0)	44.0 (1.1)	0.878
Systemic sepsis	7 (0.8)	89 (2.9)	0.001	7 (0.8)	23 (2.7)	0.006	78.5 (2.1)	103.2 (2.7)	0.607
Postoperative noninfectious complications	101 (12.0)	630 (20.8)	<0.001	101 (12.0)	180 (21.3)	<0.001	598.0 (15.6)	783.2 (20.2)	0.028
Hepatic dysfunction	42 (5.0)	192 (6.3)	0.164	42 (5.0)	54 (6.4)	0.248	263.3 (6.9)	236.2 (6.1)	0.570
Abdominal hemorrhage	6 (0.7)	79 (2.6)	0.001	6 (0.7)	19 (2.2)	0.016	54.8 (1.4)	96.3 (2.5)	0.303
Bile leak	12 (1.4)	93 (3.1)	0.013	12 (1.4)	22 (2.6)	0.119	148.6 (3.9)	111.1 (2.9)	0.382
Ascites	38 (4.5)	156 (5.1)	0.499	38 (4.5)	53 (6.3)	0.131	266.1 (7.0)	199.8 (5.2)	0.142
Pleural effusion	47 (5.6)	362 (11.9)	<0.001	47 (5.6)	102 (12.1)	<0.001	274.0 (7.2)	448.4 (11.6)	0.004
Others^*^	33 (3.9)	115 (3.8)	0.962	33 (3.9)	40 (4.7)	0.473	156.1 (4.1)	148.7 (3.8)	0.774
Postoperative hospital stay, days^†^	9.8±6.1	13.0±6.5	<0.001	9.8±6.1	13.3±7.7	<0.001	10.4±6.2	13.0±6.6	<0.001

* Others include acute pancreatitis, cardiovascular accident, upper gastrointestinal bleeding, delayed gastric emptying, deep venous thrombosis, thrombophlebitis, and wound dehiscence.

† Values are mean±SD.

IPTW, inverse probability of treatment weight; LH, laparoscopic hepatectomy; OH, open hepatectomy; PSM, propensity score matching; RI, remote infection; SSI, surgical site infection.

Regarding postoperative infectious complications, LH versus OH was associated with a lower incidence of overall infection, incisional SSI, organ/space SSI, and RI rates in the entire cohort (6.9, 1.8, 1.8, and 3.8% *vs.* 14.6, 6.3, 4.6, and 9.8%). Similar results were noted in the PSM (6.9, 1.8, 1.8, and 3.8% *vs.* 18.5, 8.4, 5.2, and 12.8%) and IPTW (9.5, 2.3, 2.1, and 5.5% *vs.* 14.3, 6.3, 4.5, and 9.8%) cohorts, respectively.

### Predictors of postoperative infectious complications

Univariate and multivariate logistic regression analyses to identify factors associated with postoperative overall infection, incisional SSI, organ/space SSI, and RI in the overall, PSM, and IPTW cohorts are shown in Supplementary Table 1–12, Supplemental Digital Content 2, http://links.lww.com/JS9/A465, Supplemental Digital Content 3, http://links.lww.com/JS9/A466, Supplemental Digital Content 4, http://links.lww.com/JS9/A467, Supplemental Digital Content 5, http://links.lww.com/JS9/A468, Supplemental Digital Content 6, http://links.lww.com/JS9/A469, Supplemental Digital Content 7, http://links.lww.com/JS9/A470, Supplemental Digital Content 8, http://links.lww.com/JS9/A471, Supplemental Digital Content 9, http://links.lww.com/JS9/A472, Supplemental Digital Content 10, http://links.lww.com/JS9/A473, Supplemental Digital Content 11, http://links.lww.com/JS9/A474, Supplemental Digital Content 12, http://links.lww.com/JS9/A475, Supplemental Digital Content 13, http://links.lww.com/JS9/A476, respectively. Table 4 depicts the odd ratios and corresponding 95% CIs based on multivariate analyses in various analytic cohorts stratified by operative approach (LH *vs.* OH). LH was an independently associated with a decreased risk of postoperative overall infection, incisional SSI, organ/space SSI, and RI.

**Table 4:** Independent effects of LH on various postoperative infectious complications by multivariate logistic regression analyses based on various analytic cohorts.

	Entire cohort		PSM cohort		IPTW cohort	
Endpoints	OR^*^ (95% CI)	*P*	OR^*^ (95% CI)	*P*	OR^*^ (95% CI)	*P*
**Overall infection**	0.45 (0.33–0.62)	<0.001	0.29 (0.21–0.41)	<0.001	0.56 (0.48–0.66)	<0.001
**Incisional SSI**	0.29 (0.17–0.50)	<0.001	0.19 (0.10–0.33)	<0.001	0.34 (0.26–0.44)	<0.001
**Organ/space SSI**	0.42 (0.24–0.74)	0.003	0.32 (0.17–0.59)	<0.001	0.38 (0.28–0.50)	<0.001
**RI**	0.36 (0.25–0.54)	<0.001	0.25 (0.17–0.39)	<0.001	0.47 (0.39–0.57)	<0.001

* Odds ratios are for the LH group, compared with the OH group.

IPTW, inverse probability of treatment weight; LH, laparoscopic hepatectomy; OH, open hepatectomy; OR, odds ratio; PSM, propensity score matching; RI, remote infection; SSI, surgical site infection.

## Discussion

Previous data have demonstrated that posthepatectomy infectious complications not only prolong hospital stay and increase hospital costs, but also compromise long-term oncological survival among patients with HCC^17,18^. The rapid development of laparoscopic techniques in hepatobiliary surgery has opened new means to reduce posthepatectomy complications. The impact of LH to reduce postoperative infectious complications following hepatectomy for HCC remains unclear, however. The current study was important because, using two propensity score methods (PSM and IPTW) and multivariate logistic regression analyses, we used a large-scale multicenter database to demonstrate that LH was an independent protective factor to reduce postoperative infectious complications following hepatectomy for HCC. Of note, LH was associated with not only a decrease in overall infection, but also lower rates of incisional SSI, organ/space SSI, and RI. As such, given the negative effect of postoperative infectious complications on long-term oncologic survival, a LH approach may be more beneficial for patients undergoing hepatectomy for HCC if feasible.

A sufficient sample size is required to identify potential differences when comparing two treatment options if the end point of the study is a small probability event. Theoretically, LH may be associated with fewer postoperative infectious complications than OH due to its minimally invasive nature with smaller incisions^24,43^. However, the incidence of overall infection after hepatectomy was previously reported to be relatively low at 14.0–24.8% in OH and 2.0–10.4% in LH^25,27,28^. In the current study, the incidence of overall infection after hepatectomy was 14.6% in OH and 6.9% in LH. Therefore, a relatively large-sample size is required to adequately investigate the possible superiority and effectiveness given the occurrence rate of the endpoint is relatively low, and the difference between the two groups not very large. To that end, the minimum sample size to achieve statistical reliability in this study would require more than 300 cases when the total infection rate is taken as the end point of the study, or at least 2000 cases when a specific type of complication with a lower incidence is taken as the end point, such as SSI or RI. To date, most previous observational studies published in the past have failed to meet the required sample size. Rather, the present study is the largest study to date (nearly 4000 cases) to specifically investigate the risk of infectious complications after hepatectomy relative to the operative approach. In addition, we not only investigated the incidence of overall infection after hepatectomy, but also specifically analyzed the three types of infectious complications.

Despite the retrospective and observational nature of the study, several statistical methods were adopted to mitigate the impact of potential selection biases. In particular, propensity score methods is a commonly used statistical method in observational research that is considered to reduce selection biases^22,44–46^. In the current study, both the PSM and the IPTW were used to simulate a randomized clinical trial (RCT) in the real world from the perspective of statistical analysis. While a well-designed, large-sample RCT may be the optimal research method to answer the study question at hand, such a study is likely not feasible. RCT requires consumptive manpower, material resources and financial resources, and high ethical requirements. Additionally, the cost-effectiveness ratio should be fully considered if infectious complications are to be the main endpoint.

In addition to the difficulty and popularity of LH, the additional expenditures, especially the equipment cost, is also a factor for surgeons and patients when considering the surgical approach, especially in China^47^. At present, the proportion of HCC patients undergoing LH has demonstrated an upward trend due to the gradually popularized laparoscopic surgery in hospitals at all levels in China, as well as the greatly reduced cost of localized laparoscopic equipment^48^. In this multicenter study, patients underwent hepatectomy over a 12 year time span from 2010 to 2021; treatment time was divided into two periods (2010–2015 vs. 2016–2021). The proportion of LH was only 17.1% in the first period versus 25.2% in the second period. Accordingly, in the entire cohort, the incidence of postoperative overall infection, incisional SSI, organ/space SSI, and RI during the second period was 8.4, 3.4, 2.8, and 5.6%, respectively, all of which were lower than during the first period (19.1, 8.1, 5.6, and 12.4%). Future studies need to define whether these short-term benefits translated into long-term prognostic benefits.

Apart from surgical approach (LH vs. OH), this study also demonstrated that operation period, obesity, diabetes mellitus, and intraoperative blood transfusion as independent risk factors of postoperative infectious complications after hepatectomy for HCC in the entire, IPTW, and PSM cohorts. Indeed, many of these preoperative and intraoperative variables related with postoperative infectious complications have also been revealed in some previous studies^13,14,17,18^.

The current study had several limitations. This was a retrospective cohort study that had potential inherent biases. Using PSM, IPTW, as well as multivariate analysis, the efficacy of LH compared with that of OH in relation to postoperative infectious complications was confirmed by adjusting for potential confounders using various statistical techniques. In addition, the data were obtained from several general hospitals in China that routinely conduct LH, rather than a global database. As such, the results of this study need to be externally validated in a Western cohort. Variability and lack of standardization in surgical and perioperative management, as well as protocols with regard to infection control were also possible, as multiple institutions were involved. Finally, limited by the retrospective nature of data collection, the present study failed to incorporate some potentially important variables such as the volume of the future liver remnant, and others surrogates reflecting postoperative immune status, function, and actual glucose control. These variables should be incorporated into future studies. In addition, we hope to conduct targeted investigations on some subgroups, such as patients with concomitant portal hypertension, patients undergoing major hepatectomy, and patients who developed postoperative bile leaks or liver failure, which are indeed very important and warrant attention. At last, the technical challenges of LH and the potential for differences in surgical approach based on tumor location, which could impact the incidence of SSIs. Further studies that account for the impact of the anatomical site of resection and other potential confounding factors on the incidence of SSIs in LH would certainly be valuable.

In summary, LH versus OH was independently associated with a lower incidence of overall postoperative infectious complications following hepatectomy for HCC. The benefits of LH over OH included a lower risk of incisional SSI, organ/space SSI, and RI. Given that LH can significantly reduce the incidence of postoperative infection in patients with HCC undergoing hepatectomy, and that postoperative infection often has adverse effects on the short- and long-term prognosis of patients, LH may further affect the prognosis of patients with HCC undergoing hepatectomy. As such, a minimally invasive approach to hepatectomy for HCC should be increasingly considered in appropriately selected patients.

## Ethics approval and consent to participate

The study was performed in accordance with the Declaration of Helsinki and was approved by the Institutional Review Boards of First Hospital of Jilin University.

## Sources of funding

This study was supported by the National Natural Science Foundation of China (No: 81972726 and 82273074), Dawn Project Foundation of Shanghai (No: 21SG36), Shanghai Health Academic Leader Program (No. 2022XD001), and Adjunct Talent Fund of Zhejiang Provincial People’s Hospital (No: 2021-YT).

## Author’s contribution

J.-L.P., X.X., L.-L.C., C.L., H.-D.J., and Z.-Q.F. contributed equally to this work. J.-L.P., X.X., C.L., T.Y., L.-L.C., T.M.P., F.S.: conception; T.Y., J.-L.P., C.L., X.X., Z.-Q.F., H.-D.J.: study design; F.S., Z.C., G.-Y.L., T.Y.: administrative support; J.-L.P., L.-L.C., X.X., C.L., H.-D.J., Z.-Q.F., J.-D.L., M.-C.G., Y.-J.L., Y.-H.Z., X.-M.W., W.-M.G., H.W., J.L., Z.-Y.C., T.-H.C., Y.-M.Z., Z.-X.C., L.-Q.Y., Y.-K.D., M.-D.W.: data collection and acquisition; J.-L.P., L.-L.C., X.X., C.L., Z.-Q.F., T.Y.: data analysis; J.-L.P., X.X., T.Y., T.M.P., W.Y.L., G.-Y.L.: manuscript preparation; Z.C., F.S., T.M.P., W.Y.L., G.-Y.L.: critical revision; Final approval of manuscript is done by all authors.

## Conflicts of interest disclosure

None.

## Research registration unique identifying number (UIN)

1. Name of the registry: Research Registry.
2. Unique Identifying number or registration ID: researchregistry8641.
3. Hyperlink to your specific registration (must be publicly accessible and will be checked): researchregistry.com/browse-the-registry#home/registrationdetails/63d1de8e2577eb00129d8966/

## Guarantor

Guo-yue Lv, Tian Yang.

## Data availability statements

The datasets analyzed during the current study are available from the corresponding author on reasonable request.

## Potential competing interest

None.

## Role of the funder/sponsor

The funding sources had no role in the design and conduct of the study; collection, management, analysis, and interpretation of the data; preparation, review, or approval of the manuscript; and decision to submit the manuscript for publication.

## Supplementary Material
