# Supplementary material for: Postoperative infectious complications following laparoscopic versus open hepatectomy for hepatocellular carcinoma: a multicenter propensity score analysis of 3876 patients
Source: Int J Surg. 2023 May 10;109(8):2267–75. doi: 10.1097/JS9.0000000000000446 (PMC10442085; doi:10.1097/JS9.0000000000000446)
Supplement: Supplementary file 2 [file js9-109-2267-s002.docx]

**Supplementary Table 1.** Univariate and multivariate logistic regression analyses of independent risk factors associated with postoperative infectious complications after hepatectomy in the entire cohort.

| **Variables** | **OR comparison** | **UV OR (95% CI)** | **UV *P*** | **MV OR (95% CI)** | **MV *P**** |
| --- | --- | --- | --- | --- | --- |
| Surgical approach | LH *vs.* OH | 0.43 (0.32 - 0.57) | < 0.001 | 0.45 (0.33 - 0.62) | < 0.001 |
| Operation period | 2010~2015 *vs.* 2016~2021 | 2.58 (2.12 - 3.13) | < 0.001 | 2.14 (1.73 - 2.65) | < 0.001 |
| Age | > 60 *vs.* ≤ 60 years | 1.24 (1.01 - 1.52) | 0.039 | NS | 0.069 |
| Sex | Male *vs.* Female | 1.14 (0.86 - 1.52) | 0.383 |  |  |
| Obesity (BMI ≥ 30.0 kg/m^2^) | Yes *vs.* No | 4.13 (2.81 – 6.00) | < 0.001 | 4.15 (2.74 - 6.30) | < 0.001 |
| Diabetes mellitus | Yes *vs.* No | 2.39 (1.82 - 3.11) | < 0.001 | 2.29 (1.69 - 3.09) | < 0.001 |
| ASA score | > 2 *vs.* ≤ 2 | 2.32 (1.87 - 2.88) | < 0.001 | 1.93 (1.51 - 2.46) | < 0.001 |
| HBV (+) | Yes *vs.* No | 0.89 (0.68 - 1.18) | 0.410 |  |  |
| HCV (+) | Yes *vs.* No | 2.49 (1.53 - 3.91) | < 0.001 | 2.12 (1.26 - 3.56) | 0.005 |
| Cirrhosis | Yes *vs.* No | 1.54 (1.23 - 1.95) | < 0.001 | 1.37 (1.06 - 1.77) | 0.018 |
| Portal hypertension | Yes *vs.* No | 1.46 (1.19 - 1.79) | < 0.001 | NS | 0.061 |
| Child-Pugh grade | B *vs.* A | 2.32 (1.77 - 3.01) | < 0.001 | 1.55 (1.16 - 2.09) | 0.003 |
| Maximum tumor size | > 5.0 *vs.* ≤ 5.0 cm | 1.77 (1.46 - 2.14) | < 0.001 | NS | 0.256 |
| Multiple tumors | Yes *vs.* No | 1.71 (1.38 - 2.12) | < 0.001 | 1.43 (1.13 - 1.82) | 0.003 |
| Gross vascular invasion | Yes *vs.* No | 2.52 (1.96 - 3.21) | < 0.001 | 1.53 (1.14 - 2.05) | 0.004 |
| Extent of hepatectomy | Major *vs.* Minor | 1.79 (1.45 - 2.19) | < 0.001 | NS | 0.306 |
| Intraoperative blood loss | > 600 *vs.* ≤ 600 ml | 2.53 (2.06 - 3.10) | < 0.001 | NS | 0.584 |
| Intraoperative blood transfusion | Yes *vs.* No | 3.44 (2.82 - 4.19) | < 0.001 | 2.46 (1.87 - 3.25) | < 0.001 |

*The variable of surgical approach and those variables found significant at *P* < 0. 1 in univariable analyses were entered into multivariable logistic regression models.

**Abbreviations:** LH, laparoscopic hepatectomy; OH, open hepatectomy; BMI, body mass index; ASA, American Society of Anesthesiologists; HBV, hepatitis B virus; HCV, hepatitis C virus; OR, odds ratio; CI, confidence interval; UV, univariable; MV, multivariable; NS, not significant.
