# Supplementary material for: Postoperative infectious complications following laparoscopic versus open hepatectomy for hepatocellular carcinoma: a multicenter propensity score analysis of 3876 patients
Source: Int J Surg. 2023 May 10;109(8):2267–75. doi: 10.1097/JS9.0000000000000446 (PMC10442085; doi:10.1097/JS9.0000000000000446)
Supplement: Supplementary file 3 [file js9-109-2267-s003.docx]

**Supplementary Table 2.** Univariate and multivariate logistic regression analyses of independent risk factors associated with postoperative infectious complications after hepatectomy in the PSM cohort.

| **Variables** | **OR comparison** | **UV OR (95% CI)** | **UV *P*** | **MV OR (95% CI)** | **MV *P**** |
| --- | --- | --- | --- | --- | --- |
| Surgical approach | LH *vs.* OH | 0.33 (0.24 - 0.44) | < 0.001 | 0.29 (0.21 - 0.41) | < 0.001 |
| Operation period | 2010~2015 *vs.* 2016~2021 | 2.70 (2.02 - 3.62) | < 0.001 | 2.24 (1.63 - 3.08) | < 0.001 |
| Age | > 60 *vs.* ≤ 60 years | 1.06 (0.79 - 1.41) | 0.691 |  |  |
| Sex | Male *vs.* Female | 1.41 (0.94 - 2.21) | 0.112 |  |  |
| Obesity (BMI ≥ 30.0 kg/m^2^) | Yes *vs.* No | 3.47 (1.71 - 6.71) | < 0.001 | 3.05 (1.41 - 6.60) | 0.005 |
| Diabetes mellitus | Yes *vs.* No | 2.01 (1.40 - 2.84) | < 0.001 | 1.96 (1.31 - 2.93) | 0.001 |
| ASA score | > 2 *vs.* ≤ 2 | 1.81 (1.31 - 2.48) | < 0.001 | 1.55 (1.08 - 2.22) | 0.018 |
| HBV (+) | Yes *vs.* No | 1.24 (0.83 - 1.90) | 0.305 |  |  |
| HCV (+) | Yes *vs.* No | 2.69 (0.86 - 7.22) | 0.062 | NS | 0.099 |
| Cirrhosis | Yes *vs.* No | 1.68 (1.18 - 2.43) | 0.005 | 1.70 (1.15 - 2.51) | 0.008 |
| Portal hypertension | Yes *vs.* No | 0.92 (0.67 - 1.25) | 0.596 |  |  |
| Child-Pugh grade | B *vs.* A | 1.78 (1.09 - 2.80) | 0.017 | NS | 0.341 |
| Maximum tumor size | > 5.0 *vs.* ≤ 5.0 cm | 2.34 (1.74 - 3.14) | < 0.001 | 1.66 (1.16 - 2.37) | 0.006 |
| Multiple tumors | Yes *vs.* No | 1.55 (1.07 - 2.21) | 0.017 | NS | 0.111 |
| Gross vascular invasion | Yes *vs.* No | 3.05 (1.86 - 4.88) | < 0.001 | NS | 0.099 |
| Extent of hepatectomy | Major *vs.* Minor | 2.40 (1.69 - 3.37) | < 0.001 | NS | 0.241 |
| Intraoperative blood loss | > 600 *vs.* ≤ 600 ml | 3.03 (2.16 - 4.22) | < 0.001 | NS | 0.130 |
| Intraoperative blood transfusion | Yes *vs.* No | 3.59 (2.62 - 4.90) | < 0.001 | 2.13 (1.38 - 3.27) | 0.001 |

*The variable of surgical approach and those variables found significant at *P* < 0. 1 in univariable analyses were entered into multivariable logistic regression models.

**Abbreviations:** PSM, propensity score matching; LH, laparoscopic hepatectomy; OH, open hepatectomy; BMI, body mass index; ASA, American Society of Anesthesiologists; HBV, hepatitis B virus; HCV, hepatitis C virus; OR, odds ratio; CI, confidence interval; UV, univariable; MV, multivariable; NS, not significant.
