# Supplementary material for: Postoperative infectious complications following laparoscopic versus open hepatectomy for hepatocellular carcinoma: a multicenter propensity score analysis of 3876 patients
Source: Int J Surg. 2023 May 10;109(8):2267–75. doi: 10.1097/JS9.0000000000000446 (PMC10442085; doi:10.1097/JS9.0000000000000446)
Supplement: Supplementary file 4 [file js9-109-2267-s004.docx]

**Supplementary Table 3.** Univariate and multivariate logistic regression analyses of independent risk factors associated with postoperative infectious complications after hepatectomy in the IPTW cohort.

| **Variables** | **OR comparison** | **UV OR (95% CI)** | **UV *P*** | **MV OR (95% CI)** | **MV *P**** |
| --- | --- | --- | --- | --- | --- |
| Surgical approach | LH *vs.* OH | 0.63 (0.54 - 0.72) | < 0.001 | 0.56 (0.48 - 0.66) | < 0.001 |
| Operation period | 2010~2015 *vs.* 2016~2021 | 2.82 (2.45 - 3.26) | < 0.001 | 2.13 (1.82 - 2.49) | < 0.001 |
| Age | > 60 *vs.* ≤ 60 years | 1.44 (1.24 - 1.66) | < 0.001 | 1.37 (1.16 - 1.62) | < 0.001 |
| Sex | Male *vs.* Female | 1.02 (0.83 - 1.25) | 0.877 |  |  |
| Obesity (BMI ≥ 30.0 kg/m^2^) | Yes *vs.* No | 3.44 (2.62 - 4.48) | < 0.001 | 3.04 (2.23 - 4.13) | < 0.001 |
| Diabetes mellitus | Yes *vs.* No | 2.38 (1.96 - 2.87) | < 0.001 | 2.25 (1.81 - 2.79) | < 0.001 |
| ASA score | > 2 *vs.* ≤ 2 | 2.30 (1.96 - 2.69) | < 0.001 | 1.81 (1.52 - 2.17) | < 0.001 |
| HBV (+) | Yes *vs.* No | 0.96 (0.79 - 1.18) | 0.711 |  |  |
| HCV (+) | Yes *vs.* No | 2.75 (1.95 - 3.81) | < 0.001 | 2.18 (1.47 - 3.23) | < 0.001 |
| Cirrhosis | Yes *vs.* No | 1.32 (1.12 - 1.56) | 0.001 | NS | 0.106 |
| Portal hypertension | Yes *vs.* No | 1.42 (1.22 - 1.65) | < 0.001 | NS | 0.376 |
| Child-Pugh grade | B *vs.* A | 3.26 (2.73 - 3.89) | < 0.001 | 1.96 (1.59 - 2.41) | < 0.001 |
| Maximum tumor size | > 5.0 *vs.* ≤ 5.0 cm | 2.14 (1.86 - 2.47) | < 0.001 | 1.27 (1.07 - 1.51) | 0.006 |
| Multiple tumors | Yes *vs.* No | 1.63 (1.39 - 1.91) | < 0.001 | 1.31 (1.10 - 1.57) | 0.003 |
| Gross vascular invasion | Yes *vs.* No | 3.17 (2.65 - 3.78) | < 0.001 | 1.87 (1.52 - 2.32) | < 0.001 |
| Extent of hepatectomy | Major *vs.* Minor | 2.19 (1.89 - 2.53) | < 0.001 | 1.24 (1.02 - 1.49) | 0.028 |
| Intraoperative blood loss | > 600 *vs.* ≤ 600 ml | 3.42 (2.96 - 3.95) | < 0.001 | 1.52 (1.24 - 1.86) | < 0.001 |
| Intraoperative blood transfusion | Yes *vs.* No | 4.21 (3.65 - 4.86) | < 0.001 | 2.16 (1.77 - 2.64) | < 0.001 |

*The variable of surgical approach and those variables found significant at *P* < 0. 1 in univariable analyses were entered into multivariable logistic regression models.

**Abbreviations:** IPTW, inverse probability of treatment weight; LH, laparoscopic hepatectomy; OH, open hepatectomy; BMI, body mass index; ASA, American Society of Anesthesiologists; HBV, hepatitis B virus; HCV, hepatitis C virus; OR, odds ratio; CI, confidence interval; UV, univariable; MV, multivariable; NS, not significant.
