# Supplementary material for: Postoperative infectious complications following laparoscopic versus open hepatectomy for hepatocellular carcinoma: a multicenter propensity score analysis of 3876 patients
Source: Int J Surg. 2023 May 10;109(8):2267–75. doi: 10.1097/JS9.0000000000000446 (PMC10442085; doi:10.1097/JS9.0000000000000446)
Supplement: Supplementary file 5 [file js9-109-2267-s005.docx]

**Supplementary Table 4.** Univariate and multivariate logistic regression analyses of independent risk factors associated with incisional SSI after hepatectomy in the entire cohort.

| **Variables** | **OR comparison** | **UV OR (95% CI)** | **UV *P*** | **MV OR (95% CI)** | **MV *P**** |
| --- | --- | --- | --- | --- | --- |
| Surgical approach | LH *vs.* OH | 0.27 (0.15 - 0.44) | < 0.001 | 0.29 (0.17 - 0.50) | < 0.001 |
| Operation period | 2010~2015 *vs.* 2016~2021 | 2.50 (1.88 - 3.36) | < 0.001 | 1.96 (1.44 - 2.67) | < 0.001 |
| Age | > 60 *vs.* ≤ 60 years | 1.21 (0.89 - 1.63) | 0.223 |  |  |
| Sex | Male *vs.* Female | 1.32 (0.86 - 2.14) | 0.224 |  |  |
| Obesity (BMI ≥ 30.0 kg/m^2^) | Yes *vs.* No | 3.43 (2.00 - 5.59) | < 0.001 | 3.17 (1.84 - 5.44) | < 0.001 |
| Diabetes mellitus | Yes *vs.* No | 2.53 (1.73 - 3.62) | < 0.001 | 2.58 (1.73 - 3.84) | < 0.001 |
| ASA score | > 2 *vs.* ≤ 2 | 2.19 (1.59 - 2.98) | < 0.001 | 1.82 (1.30 - 2.56) | < 0.001 |
| HBV (+) | Yes *vs.* No | 0.80 (0.55 - 1.19) | 0.250 |  |  |
| HCV (+) | Yes *vs.* No | 1.66 (0.73 - 3.26) | 0.181 |  |  |
| Cirrhosis | Yes *vs.* No | 1.36 (0.98 - 1.93) | 0.077 | NS | 0.326 |
| Portal hypertension | Yes *vs.* No | 1.30 (0.95 - 1.76) | 0.092 | NS | 0.351 |
| Child-Pugh grade | B *vs.* A | 2.02 (1.36 - 2.93) | < 0.001 | NS | 0.130 |
| Maximum tumor size | > 5.0 *vs.* ≤ 5.0 cm | 1.98 (1.49 - 2.66) | < 0.001 | NS | 0.132 |
| Multiple tumors | Yes *vs.* No | 1.54 (1.11 - 2.11) | 0.008 | NS | 0.160 |
| Gross vascular invasion | Yes *vs.* No | 2.48 (1.74 - 3.49) | < 0.001 | 1.52 (1.02 - 2.27) | 0.039 |
| Extent of hepatectomy | Major *vs.* Minor | 1.62 (1.19 - 2.19) | 0.002 | NS | 0.834 |
| Intraoperative blood loss | > 600 *vs.* ≤ 600 ml | 2.34 (1.73 - 3.13) | < 0.001 | NS | 0.526 |
| Intraoperative blood transfusion | Yes *vs.* No | 3.15 (2.36 - 4.19) | < 0.001 | 2.33 (1.57 - 3.46) | < 0.001 |

*The variable of surgical approach and those variables found significant at *P* < 0. 1 in univariable analyses were entered into multivariable logistic regression models.

**Abbreviations:** SSI, surgical site infection; LH, laparoscopic hepatectomy; OH, open hepatectomy; BMI, body mass index; ASA, American Society of Anesthesiologists; HBV, hepatitis B virus; HCV, hepatitis C virus; OR, odds ratio; CI, confidence interval; UV, univariable; MV, multivariable; NS, not significant.
