# Supplementary material for: Postoperative infectious complications following laparoscopic versus open hepatectomy for hepatocellular carcinoma: a multicenter propensity score analysis of 3876 patients
Source: Int J Surg. 2023 May 10;109(8):2267–75. doi: 10.1097/JS9.0000000000000446 (PMC10442085; doi:10.1097/JS9.0000000000000446)
Supplement: Supplementary file 6 [file js9-109-2267-s006.docx]

**Supplementary Table 5.** Univariate and multivariate logistic regression analyses of independent risk factors associated with incisional SSI after hepatectomy in the PSM cohort.

| **Variables** | **OR comparison** | **UV OR (95% CI)** | **UV *P*** | **MV OR (95% CI)** | **MV *P**** |
| --- | --- | --- | --- | --- | --- |
| Surgical approach | LH *vs.* OH | 0.20 (0.11 - 0.34) | < 0.001 | 0.19 (0.10 - 0.33) | < 0.001 |
| Operation period | 2010~2015 *vs.* 2016~2021 | 2.33 (1.50 - 3.61) | < 0.001 | 1.95 (1.22 - 3.13) | 0.005 |
| Age | > 60 *vs.* ≤ 60 years | 0.99 (0.64 - 1.54) | 0.983 |  |  |
| Sex | Male *vs.* Female | 1.52 (0.81 - 3.16) | 0.224 |  |  |
| Obesity (BMI ≥ 30.0 kg/m^2^) | Yes *vs.* No | 3.46 (1.28 - 7.93) | 0.007 | 3.00 (1.13 - 8.00) | 0.028 |
| Diabetes mellitus | Yes *vs.* No | 3.00 (1.84 - 4.80) | < 0.001 | 2.88 (1.70 - 4.87) | < 0.001 |
| ASA score | > 2 *vs.* ≤ 2 | 1.84 (1.13 - 2.92) | 0.011 | 1.70 (1.05 -2.77) | 0.032 |
| HBV (+) | Yes *vs.* No | 1.11 (0.63 - 2.13) | 0.732 |  |  |
| HCV (+) | Yes *vs.* No | 1.10 (0.06 - 5.44) | 0.928 |  |  |
| Cirrhosis | Yes *vs.* No | 1.17 (0.72 - 1.98) | 0.541 |  |  |
| Portal hypertension | Yes *vs.* No | 0.71 (0.42 - 1.15) | 0.177 |  |  |
| Child-Pugh grade | B *vs.* A | 1.34 (0.58 - 2.68) | 0.445 |  |  |
| Maximum tumor size | > 5.0 *vs.* ≤ 5.0 cm | 2.92 (1.88 - 4.53) | < 0.001 | 2.36 (1.44 - 3.89) | 0.001 |
| Multiple tumors | Yes *vs.* No | 1.64 (0.95 - 2.73) | 0.065 | NS | 0.152 |
| Gross vascular invasion | Yes *vs.* No | 1.36 (0.52 - 2.96) | 0.486 |  |  |
| Extent of hepatectomy | Major *vs.* Minor | 1.81 (1.04 - 3.02) | 0.028 | NS | 0.588 |
| Intraoperative blood loss | > 600 *vs.* ≤ 600 ml | 3.04 (1.86 - 4.85) | < 0.001 | 2.11 (1.09 - 4.12) | 0.028 |
| Intraoperative blood transfusion | Yes *vs.* No | 2.86 (1.79 - 4.50) | < 0.001 | NS | 0.335 |

*The variable of surgical approach and those variables found significant at *P* < 0. 1 in univariable analyses were entered into multivariable logistic regression models.

**Abbreviations:** SSI, surgical site infection; PSM, propensity score matching; LH, laparoscopic hepatectomy; OH, open hepatectomy; BMI, body mass index; ASA, American Society of Anesthesiologists; HBV, hepatitis B virus; HCV, hepatitis C virus; OR, odds ratio; CI, confidence interval; UV, univariable; MV, multivariable; NS, not significant.
