# Supplementary material for: Postoperative infectious complications following laparoscopic versus open hepatectomy for hepatocellular carcinoma: a multicenter propensity score analysis of 3876 patients
Source: Int J Surg. 2023 May 10;109(8):2267–75. doi: 10.1097/JS9.0000000000000446 (PMC10442085; doi:10.1097/JS9.0000000000000446)
Supplement: Supplementary file 7 [file js9-109-2267-s007.docx]

**Supplementary Table 6.** Univariate and multivariate logistic regression analyses of independent risk factors associated with incisional SSI after hepatectomy in the IPTW cohort.

| **Variables** | **OR comparison** | **UV OR (95% CI)** | **UV *P*** | **MV OR (95% CI)** | **MV *P**** |
| --- | --- | --- | --- | --- | --- |
| Surgical approach | LH *vs.* OH | 0.35 (0.28 - 0.45) | < 0.001 | 0.34 (0.26 - 0.44) | < 0.001 |
| Operation period | 2010~2015 *vs.* 2016~2021 | 2.21 (1.77 - 2.77) | < 0.001 | 1.80 (1.42 - 2.27) | < 0.001 |
| Age | > 60 *vs.* ≤ 60 years | 1.32 (1.04 - 1.67) | 0.019 | NS | 0.130 |
| Sex | Male *vs.* Female | 0.96 (0.71 - 1.34) | 0.817 |  |  |
| Obesity (BMI ≥ 30.0 kg/m^2^) | Yes *vs.* No | 2.84 (1.86 - 4.17) | < 0.001 | 2.63 (1.72 - 4.03) | < 0.001 |
| Diabetes mellitus | Yes *vs.* No | 2.43 (1.81 - 3.20) | < 0.001 | 2.23 (1.64 - 3.03) | < 0.001 |
| ASA score | > 2 *vs.* ≤ 2 | 2.03 (1.58 - 2.59) | < 0.001 | 1.68 (1.28 - 2.19) | < 0.001 |
| HBV (+) | Yes *vs.* No | 0.86 (0.64 - 1.19) | 0.356 |  |  |
| HCV (+) | Yes *vs.* No | 1.14 (0.54 - 2.12) | 0.699 |  |  |
| Cirrhosis | Yes *vs.* No | 1.10 (0.86 - 1.44) | 0.445 |  |  |
| Portal hypertension | Yes *vs.* No | 1.39 (1.09 - 1.75) | 0.007 | 1.34 (1.04 - 1.74) | 0.026 |
| Child-Pugh grade | B *vs.* A | 1.37 (0.98 - 1.89) | 0.058 | NS | 0.541 |
| Maximum tumor size | > 5.0 *vs.* ≤ 5.0 cm | 1.88 (1.50 - 2.36) | < 0.001 | 1.51 (1.16 - 1.95) | 0.002 |
| Multiple tumors | Yes *vs.* No | 1.48 (1.15 - 1.90) | 0.002 | NS | 0.051 |
| Gross vascular invasion | Yes *vs.* No | 1.71 (1.25 - 2.31) | < 0.001 | NS | 0.894 |
| Extent of hepatectomy | Major *vs.* Minor | 1.48 (1.15 - 1.87) | 0.002 | NS | 0.656 |
| Intraoperative blood loss | > 600 *vs.* ≤ 600 ml | 2.83 (2.25 - 3.54) | < 0.001 | 1.93 (1.41 - 2.64) | < 0.001 |
| Intraoperative blood transfusion | Yes *vs.* No | 2.70 (2.15 - 3.38) | < 0.001 | 1.38 (1.01 - 1.89) | 0.043 |

*The variable of surgical approach and those variables found significant at *P* < 0. 1 in univariable analyses were entered into multivariable logistic regression models.

**Abbreviations:** SSI, surgical site infection; IPTW, inverse probability of treatment weight; LH, laparoscopic hepatectomy; OH, open hepatectomy; BMI, body mass index; ASA, American Society of Anesthesiologists; HBV, hepatitis B virus; HCV, hepatitis C virus; OR, odds ratio; CI, confidence interval; UV, univariable; MV, multivariable; NS, not significant.
