# Supplementary material for: Postoperative infectious complications following laparoscopic versus open hepatectomy for hepatocellular carcinoma: a multicenter propensity score analysis of 3876 patients
Source: Int J Surg. 2023 May 10;109(8):2267–75. doi: 10.1097/JS9.0000000000000446 (PMC10442085; doi:10.1097/JS9.0000000000000446)
Supplement: Supplementary file 8 [file js9-109-2267-s008.docx]

**Supplementary Table 7.** Univariate and multivariate logistic regression analyses of independent risk factors associated with organ/space SSI after hepatectomy in the entire cohort.

| **Variables** | **OR comparison** | **UV OR (95% CI)** | **UV *P*** | **MV OR (95% CI)** | **MV *P**** |
| --- | --- | --- | --- | --- | --- |
| Surgical approach | LH *vs.* OH | 0.37 (0.21 - 0.62) | < 0.001 | 0.42 (0.24 - 0.74) | 0.003 |
| Operation period | 2010~2015 *vs.* 2016~2021 | 2.02 (1.46 - 2.81) | < 0.001 | 1.52 (1.07 - 2.17) | 0.019 |
| Age | > 60 *vs.* ≤ 60 years | 1.12 (0.78 - 1.59) | 0.518 |  |  |
| Sex | Male *vs.* Female | 1.05 (0.67 - 1.75) | 0.841 |  |  |
| Obesity (BMI ≥ 30.0 kg/m^2^) | Yes *vs.* No | 4.16 (2.35 - 6.96) | < 0.001 | 3.46 (1.93 - 6.21) | < 0.001 |
| Diabetes mellitus | Yes *vs.* No | 3.19 (2.12 - 4.68) | < 0.001 | 3.29 (2.12 - 5.10) | < 0.001 |
| ASA score | > 2 *vs.* ≤ 2 | 2.45 (1.71 - 3.46) | < 0.001 | 1.98 (1.35 - 2.89) | < 0.001 |
| HBV (+) | Yes *vs.* No | 1.21 (0.74 - 2.08) | 0.476 |  |  |
| HCV (+) | Yes *vs.* No | 2.27 (1.08 - 4.78) | 0.030 | NS | 0.301 |
| Cirrhosis | Yes *vs.* No | 2.67 (1.68 - 4.47) | < 0.001 | 2.38 (1.43 - 3.97) | 0.001 |
| Portal hypertension | Yes *vs.* No | 1.74 (1.24 - 2.43) | 0.001 | NS | 0.318 |
| Child-Pugh grade | B *vs.* A | 2.94 (1.95 - 4.32) | < 0.001 | 1.89 (1.23 - 2.92) | 0.004 |
| Maximum tumor size | > 5.0 *vs.* ≤ 5.0 cm | 2.00 (1.44 - 2.80) | < 0.001 | NS | 0.357 |
| Multiple tumors | Yes *vs.* No | 1.74 (1.21 - 2.47) | 0.002 | NS | 0.206 |
| Gross vascular invasion | Yes *vs.* No | 3.11 (2.12 - 4.49) | < 0.001 | 1.66 (1.07 - 2.58) | 0.023 |
| Extent of hepatectomy | Major *vs.* Minor | 2.18 (1.55 - 3.04) | < 0.001 | NS | 0.122 |
| Intraoperative blood loss | > 600 *vs.* ≤ 600 ml | 3.11 (2.23 - 4.31) | < 0.001 | NS | 0.439 |
| Intraoperative blood transfusion | Yes *vs.* No | 3.53 (2.55 - 4.89) | < 0.001 | 2.01 (1.27 - 3.18) | 0.003 |

*The variable of surgical approach and those variables found significant at *P* < 0. 1 in univariable analyses were entered into multivariable logistic regression models.

**Abbreviations:** SSI, surgical site infection; LH, laparoscopic hepatectomy; OH, open hepatectomy; BMI, body mass index; ASA, American Society of Anesthesiologists; HBV, hepatitis B virus; HCV, hepatitis C virus; OR, odds ratio; CI, confidence interval; UV, univariable; MV, multivariable; NS, not significant.
