# Supplementary material for: Postoperative infectious complications following laparoscopic versus open hepatectomy for hepatocellular carcinoma: a multicenter propensity score analysis of 3876 patients
Source: Int J Surg. 2023 May 10;109(8):2267–75. doi: 10.1097/JS9.0000000000000446 (PMC10442085; doi:10.1097/JS9.0000000000000446)
Supplement: Supplementary file 9 [file js9-109-2267-s009.docx]

**Supplementary Table 8.** Univariate and multivariate logistic regression analyses of independent risk factors associated with organ/space SSI after hepatectomy in the PSM cohort.

| **Variables** | **OR comparison** | **UV OR (95% CI)** | **UV *P*** | **MV OR (95% CI)** | **MV *P**** |
| --- | --- | --- | --- | --- | --- |
| Surgical approach | LH *vs.* OH | 0.33 (0.18 - 0.58) | < 0.001 | 0.32 (0.17 - 0.59) | < 0.001 |
| Operation period | 2010~2015 *vs.* 2016~2021 | 2.31 (1.37 - 3.91) | 0.002 | 1.77 (1.04 - 3.01) | 0.034 |
| Age | > 60 *vs.* ≤ 60 years | 1.29 (0.76 - 2.17) | 0.343 |  |  |
| Sex | Male *vs.* Female | 1.09 (0.56 - 2.40) | 0.810 |  |  |
| Obesity (BMI ≥ 30.0 kg/m^2^) | Yes *vs.* No | 4.22 (1.41 - 10.31) | 0.004 | 3.29 (1.08 - 10.02) | 0.036 |
| Diabetes mellitus | Yes *vs.* No | 2.80 (1.55 - 4.89) | < 0.001 | 2.60 (1.36 - 4.97) | 0.004 |
| ASA score | > 2 *vs.* ≤ 2 | 2.22 (1.27 - 3.80) | 0.004 | 1.99 (1.11 – 3.57) | 0.021 |
| HBV (+) | Yes *vs.* No | 1.26 (0.63 - 2.91) | 0.543 |  |  |
| HCV (+) | Yes *vs.* No | 3.54 (0.55 - 12.85) | 0.097 | NS | 0.257 |
| Cirrhosis | Yes *vs.* No | 3.42 (1.58 - 8.94) | 0.005 | 3.45 (1.43 - 8.35) | 0.006 |
| Portal hypertension | Yes *vs.* No | 1.55 (0.90 - 2.62) | 0.105 |  |  |
| Child-Pugh grade | B *vs.* A | 1.77 (0.72 - 3.75) | 0.166 |  |  |
| Maximum tumor size | > 5.0 *vs.* ≤ 5.0 cm | 2.57 (1.52 - 4.34) | < 0.001 | NS | 0.198 |
| Multiple tumors | Yes *vs.* No | 2.17 (1.17 - 3.83) | 0.010 | NS | 0.067 |
| Gross vascular invasion | Yes *vs.* No | 3.44 (1.54 - 6.93) | 0.001 | NS | 0.529 |
| Extent of hepatectomy | Major *vs.* Minor | 3.10 (1.73 - 5.37) | < 0.001 | NS | 0.171 |
| Intraoperative blood loss | > 600 *vs.* ≤ 600 ml | 4.56 (2.64 - 7.79) | < 0.001 | NS | 0.182 |
| Intraoperative blood transfusion | Yes *vs.* No | 5.33 (3.14 - 9.06) | < 0.001 | 2.81 (1.35 - 5.83) | 0.006 |

*The variable of surgical approach and those variables found significant at *P* < 0. 1 in univariable analyses were entered into multivariable logistic regression models.

**Abbreviations:** SSI, surgical site infection; PSM, propensity score matching; LH, laparoscopic hepatectomy; OH, open hepatectomy; BMI, body mass index; ASA, American Society of Anesthesiologists; HBV, hepatitis B virus; HCV, hepatitis C virus; OR, odds ratio; CI, confidence interval; UV, univariable; MV, multivariable; NS, not significant.
