# Supplementary material for: Postoperative infectious complications following laparoscopic versus open hepatectomy for hepatocellular carcinoma: a multicenter propensity score analysis of 3876 patients
Source: Int J Surg. 2023 May 10;109(8):2267–75. doi: 10.1097/JS9.0000000000000446 (PMC10442085; doi:10.1097/JS9.0000000000000446)
Supplement: Supplementary file 10 [file js9-109-2267-s010.docx]

**Supplementary Table 9.** Univariate and multivariate logistic regression analyses of independent risk factors associated with organ/space SSI after hepatectomy in the IPTW cohort.

| **Variables** | **OR comparison** | **UV OR (95% CI)** | **UV *P*** | **MV OR (95% CI)** | **MV *P**** |
| --- | --- | --- | --- | --- | --- |
| Surgical approach | LH *vs.* OH | 0.47 (0.36 - 0.61) | < 0.001 | 0.38 (0.28 - 0.50) | < 0.001 |
| Operation period | 2010~2015 *vs.* 2016~2021 | 2.97 (2.28 - 3.88) | < 0.001 | 2.12 (1.60 - 2.83) | < 0.001 |
| Age | > 60 *vs.* ≤ 60 years | 2.25 (1.75 - 2.89) | < 0.001 | 1.88 (1.41 - 2.50) | < 0.001 |
| Sex | Male *vs.* Female | 1.17 (0.81 - 1.76) | 0.429 |  |  |
| Obesity (BMI ≥ 30.0 kg/m^2^) | Yes *vs.* No | 6.05 (4.18 - 8.57) | < 0.001 | 4.44 (2.86 - 6.91) | < 0.001 |
| Diabetes mellitus | Yes *vs.* No | 4.05 (3.03 - 5.36) | < 0.001 | 2.81 (2.01 - 3.94) | < 0.001 |
| ASA score | > 2 *vs.* ≤ 2 | 2.74 (2.09 - 3.56) | < 0.001 | 2.06 (1.54 - 2.77) | < 0.001 |
| HBV (+) | Yes *vs.* No | 0.76 (0.55 - 1.08) | 0.113 |  |  |
| HCV (+) | Yes *vs.* No | 6.23 (4.06 - 9.28) | < 0.001 | 3.19 (1.87 - 5.42) | < 0.001 |
| Cirrhosis | Yes *vs.* No | 3.10 (2.11 - 4.74) | < 0.001 | 3.00 (1.96 - 4.59) | < 0.001 |
| Portal hypertension | Yes *vs.* No | 1.80 (1.38 - 2.32) | < 0.001 | NS | 0.239 |
| Child-Pugh grade | B *vs.* A | 2.89 (2.12 - 3.87) | < 0.001 | 1.50(1.05 - 2.13) | 0.026 |
| Maximum tumor size | > 5.0 *vs.* ≤ 5.0 cm | 2.27 (1.75 - 2.97) | < 0.001 | 1.60 (1.18 - 2.18) | 0.003 |
| Multiple tumors | Yes *vs.* No | 1.23 (0.90 - 1.64) | 0.183 |  |  |
| Gross vascular invasion | Yes *vs.* No | 2.62 (1.91 - 3.54) | < 0.001 | 1.64 (1.14 - 2.34) | 0.007 |
| Extent of hepatectomy | Major *vs.* Minor | 1.62 (1.23 - 2.12) | < 0.001 | NS | 0.396 |
| Intraoperative blood loss | > 600 *vs.* ≤ 600 ml | 2.27 (1.74 - 2.94) | < 0.001 | NS | 0.883 |
| Intraoperative blood transfusion | Yes *vs.* No | 3.37 (2.62 - 4.33) | < 0.001 | 2.42 (1.70 - 3.43) | < 0.001 |

*The variable of surgical approach and those variables found significant at *P* < 0. 1 in univariable analyses were entered into multivariable logistic regression models.

**Abbreviations:** SSI, surgical site infection; IPTW, inverse probability of treatment weight; LH, laparoscopic hepatectomy; OH, open hepatectomy; BMI, body mass index; ASA, American Society of Anesthesiologists; HBV, hepatitis B virus; HCV, hepatitis C virus; OR, odds ratio; CI, confidence interval; UV, univariable; MV, multivariable; NS, not significant.
