# Supplementary material for: Postoperative infectious complications following laparoscopic versus open hepatectomy for hepatocellular carcinoma: a multicenter propensity score analysis of 3876 patients
Source: Int J Surg. 2023 May 10;109(8):2267–75. doi: 10.1097/JS9.0000000000000446 (PMC10442085; doi:10.1097/JS9.0000000000000446)
Supplement: Supplementary file 11 [file js9-109-2267-s011.docx]

**Supplementary Table 10.** Univariate and multivariate logistic regression analyses of independent risk factors associated with RI after hepatectomy in the entire cohort.

| **Variables** | **OR comparison** | **UV OR (95% CI)** | **UV *P*** | **MV OR (95% CI)** | **MV *P**** |
| --- | --- | --- | --- | --- | --- |
| Surgical approach | LH *vs.* OH | 0.36 (0.24 - 0.52) | < 0.001 | 0.36 (0.25 - 0.54) | < 0.001 |
| Operation period | 2010~2015 *vs.* 2016~2021 | 2.37 (1.88 - 2.99) | < 0.001 | 1.90 (1.48 - 2.44) | < 0.001 |
| Age | > 60 *vs.* ≤ 60 years | 1.18 (0.92 - 1.50) | 0.198 |  |  |
| Sex | Male *vs.* Female | 1.13 (0.81 - 1.62) | 0.476 |  |  |
| Obesity (BMI ≥ 30.0 kg/m^2^) | Yes *vs.* No | 2.71 (1.68 - 4.23) | < 0.001 | 2.34 (1.42 - 3.84) | 0.001 |
| Diabetes mellitus | Yes *vs.* No | 2.66 (1.95 - 3.57) | < 0.001 | 2.68 (1.92 - 3.75) | < 0.001 |
| ASA score | > 2 *vs.* ≤ 2 | 2.36 (1.82 - 3.03) | < 0.001 | 1.95 (1.48 - 2.58) | < 0.001 |
| HBV (+) | Yes *vs.* No | 1.07 (0.77 - 1.53) | 0.696 |  |  |
| HCV (+) | Yes *vs.* No | 2.61 (1.50 - 4.31) | < 0.001 | 2.15 (1.21 - 3.82) | 0.009 |
| Cirrhosis | Yes *vs.* No | 1.55 (1.17 - 2.07) | 0.002 | NS | 0.081 |
| Portal hypertension | Yes *vs.* No | 1.57 (1.23 - 2.00) | < 0.001 | 1.35 (1.02 - 1.78) | 0.036 |
| Child-Pugh grade | B *vs.* A | 2.42 (1.78 - 3.26) | < 0.001 | 1.59 (1.14 - 2.22) | 0.007 |
| Maximum tumor size | > 5.0 *vs.* ≤ 5.0 cm | 1.57 (1.25 - 1.97) | < 0.001 | NS | 0.837 |
| Multiple tumors | Yes *vs.* No | 1.64 (1.26 - 2.11) | < 0.001 | 1.34 (1.02 - 1.77) | 0.037 |
| Gross vascular invasion | Yes *vs.* No | 2.21 (1.63 - 2.94) | < 0.001 | NS | 0.157 |
| Extent of hepatectomy | Major *vs.* Minor | 1.82 (1.42 - 2.32) | < 0.001 | NS | 0.102 |
| Intraoperative blood loss | > 600 *vs.* ≤ 600 ml | 2.57 (2.02 - 3.27) | < 0.001 | NS | 0.630 |
| Intraoperative blood transfusion | Yes *vs.* No | 3.53 (2.80 - 4.46) | < 0.001 | 2.61 (1.89 - 3.61) | < 0.001 |

*The variable of surgical approach and those variables found significant at *P* < 0. 1 in univariable analyses were entered into multivariable logistic regression models.

**Abbreviations:** RI, remote infection; LH, laparoscopic hepatectomy; OH, open hepatectomy; BMI, body mass index; ASA, American Society of Anesthesiologists; HBV, hepatitis B virus; HCV, hepatitis C virus; OR, odds ratio; CI, confidence interval; UV, univariable; MV, multivariable; NS, not significant.
