# Supplementary material for: Postoperative infectious complications following laparoscopic versus open hepatectomy for hepatocellular carcinoma: a multicenter propensity score analysis of 3876 patients
Source: Int J Surg. 2023 May 10;109(8):2267–75. doi: 10.1097/JS9.0000000000000446 (PMC10442085; doi:10.1097/JS9.0000000000000446)
Supplement: Supplementary file 12 [file js9-109-2267-s012.docx]

**Supplementary Table 11.** Univariate and multivariate logistic regression analyses of independent risk factors associated with RI after hepatectomy in the PSM cohort.

| **Variables** | **OR comparison** | **UV OR (95% CI)** | **UV *P*** | **MV OR (95% CI)** | **MV *P**** |
| --- | --- | --- | --- | --- | --- |
| Surgical approach | LH *vs.* OH | 0.27 (0.18 - 0.40) | < 0.001 | 0.25 (0.17 - 0.39) | < 0.001 |
| Operation period | 2010~2015 *vs.* 2016~2021 | 2.24 (1.58 - 3.18) | < 0.001 | 1.90 (1.30 - 2.77) | 0.001 |
| Age | > 60 *vs.* ≤ 60 years | 0.93 (0.65 - 1.31) | 0.670 |  |  |
| Sex | Male *vs.* Female | 1.57 (0.94 - 2.79) | 0.101 |  |  |
| Obesity (BMI ≥ 30.0 kg/m^2^) | Yes *vs.* No | 2.42 (1.05 - 5.57) | 0.038 | 2.52 (1.03 – 6.19) | 0.043 |
| Diabetes mellitus | Yes *vs.* No | 2.01 (1.31 - 3.02) | 0.001 | 2.26 (1.42 - 3.59) | 0.001 |
| ASA score | > 2 *vs.* ≤ 2 | 1.63 (1.09 - 2.38) | 0.014 | 1.56 (1.04 – 2.35) | 0.033 |
| HBV (+) | Yes *vs.* No | 1.70 (1.01 - 3.08) | 0.059 | NS | 0.077 |
| HCV (+) | Yes *vs.* No | 2.24 (0.51 - 6.89) | 0.206 |  |  |
| Cirrhosis | Yes *vs.* No | 2.00 (1.29 - 3.25) | 0.003 | 1.68 (1.01 - 2.78) | 0.044 |
| Portal hypertension | Yes *vs.* No | 1.26 (0.87 - 1.80) | 0.209 |  |  |
| Child-Pugh grade | B *vs.* A | 1.90 (1.07 - 3.20) | 0.021 | NS | 0.232 |
| Maximum tumor size | > 5.0 *vs.* ≤ 5.0 cm | 1.64 (1.14 - 2.35) | 0.007 | NS | 0.893 |
| Multiple tumors | Yes *vs.* No | 1.46 (0.93 - 2.23) | 0.092 | NS | 0.334 |
| Gross vascular invasion | Yes *vs.* No | 3.27 (1.86 - 5.50) | < 0.001 | 1.90 (1.01 - 3.59) | 0.048 |
| Extent of hepatectomy | Major *vs.* Minor | 2.44 (1.61 - 3.62) | < 0.001 | 1.72 (1.04 - 2.86) | 0.036 |
| Intraoperative blood loss | > 600 *vs.* ≤ 600 ml | 2.86 (1.91 - 4.21) | < 0.001 | NS | 0.281 |
| Intraoperative blood transfusion | Yes *vs.* No | 3.29 (2.27 - 4.74) | < 0.001 | 2.04 (1.23 - 3.39) | 0.006 |

*The variable of surgical approach and those variables found significant at *P* < 0. 1 in univariable analyses were entered into multivariable logistic regression models.

**Abbreviations:** RI, remote infection; PSM, propensity score matching; LH, laparoscopic hepatectomy; OH, open hepatectomy; BMI, body mass index; ASA, American Society of Anesthesiologists; HBV, hepatitis B virus; HCV, hepatitis C virus; OR, odds ratio; CI, confidence interval; UV, univariable; MV, multivariable; NS, not significant.
