# Supplementary material for: Postoperative infectious complications following laparoscopic versus open hepatectomy for hepatocellular carcinoma: a multicenter propensity score analysis of 3876 patients
Source: Int J Surg. 2023 May 10;109(8):2267–75. doi: 10.1097/JS9.0000000000000446 (PMC10442085; doi:10.1097/JS9.0000000000000446)
Supplement: Supplementary file 13 [file js9-109-2267-s013.docx]

**Supplementary Table 12.** Univariate and multivariate logistic regression analyses of independent risk factors associated with RI after hepatectomy in the IPTW cohort.

| **Variables** | **OR comparison** | **UV OR (95% CI)** | **UV *P*** | **MV OR (95% CI)** | **MV *P**** |
| --- | --- | --- | --- | --- | --- |
| Surgical approach | LH *vs.* OH | 0.54 (0.45 - 0.64) | < 0.001 | 0.47 (0.39 - 0.57) | < 0.001 |
| Operation period | 2010~2015 *vs.* 2016~2021 | 2.42 (2.04 - 2.87) | < 0.001 | 1.80 (1.49 - 2.17) | < 0.001 |
| Age | > 60 *vs.* ≤ 60 years | 1.12 (0.93 - 1.34) | 0.242 |  |  |
| Sex | Male *vs.* Female | 1.05 (0.82 - 1.36) | 0.728 |  |  |
| Obesity (BMI ≥ 30.0 kg/m^2^) | Yes *vs.* No | 1.73 (1.17 - 2.48) | 0.004 | 1.54 (1.02 - 2.32) | 0.040 |
| Diabetes mellitus | Yes *vs.* No | 2.37 (1.89 - 2.96) | < 0.001 | 2.79 (2.17 - 3.59) | < 0.001 |
| ASA score | > 2 *vs.* ≤ 2 | 2.24 (1.85 - 2.70) | < 0.001 | 1.73 (1.41 - 2.14) | < 0.001 |
| HBV (+) | Yes *vs.* No | 1.33 (1.02 - 1.77) | 0.040 | NS | 0.054 |
| HCV (+) | Yes *vs.* No | 1.52 (0.92 - 2.36) | 0.080 | NS | 0.057 |
| Cirrhosis | Yes *vs.* No | 1.20 (0.99 - 1.47) | 0.073 | NS | 0.402 |
| Portal hypertension | Yes *vs.* No | 1.44 (1.20 - 1.72) | < 0.001 | NS | 0.647 |
| Child-Pugh grade | B *vs.* A | 3.81 (3.11 - 4.65) | < 0.001 | 2.36 (1.87 - 2.98) | < 0.001 |
| Maximum tumor size | > 5.0 *vs.* ≤ 5.0 cm | 1.74 (1.47 - 2.06) | < 0.001 | NS | 0.052 |
| Multiple tumors | Yes *vs.* No | 1.74 (1.43 - 2.09) | < 0.001 | 1.30 (1.05 - 1.61) | 0.015 |
| Gross vascular invasion | Yes *vs.* No | 3.60 (2.94 - 4.41) | < 0.001 | 1.99 (1.56 - 2.53) | < 0.001 |
| Extent of hepatectomy | Major *vs.* Minor | 2.75 (2.31 - 3.27) | < 0.001 | 1.69 (1.35 - 2.12) | < 0.001 |
| Intraoperative blood loss | > 600 *vs.* ≤ 600 ml | 3.82 (3.22 - 4.54) | < 0.001 | 1.35 (1.06 - 1.73) | 0.016 |
| Intraoperative blood transfusion | Yes *vs.* No | 5.01 (4.22 - 5.95) | < 0.001 | 2.78 (2.18 - 3.53) | < 0.001 |

*The variable of surgical approach and those variables found significant at *P* < 0. 1 in univariable analyses were entered into multivariable logistic regression models.

**Abbreviations:** RI, remote infection; IPTW, inverse probability of treatment weight; LH, laparoscopic hepatectomy; OH, open hepatectomy; BMI, body mass index; ASA, American Society of Anesthesiologists; HBV, hepatitis B virus; HCV, hepatitis C virus; OR, odds ratio; CI, confidence interval; UV, univariable; MV, multivariable; NS, not significant.
